# A validation study of the European Society of Cardiology guidelines for risk stratification of sudden cardiac death in childhood hypertrophic cardiomyopathy

**DOI:** 10.1093/europace/euz118

**Published:** 2019-06-01

**Authors:** Gabrielle Norrish, Tao Ding, Ella Field, Karen McLeod, Maria Ilina, Graham Stuart, Vinay Bhole, Orhan Uzun, Elspeth Brown, Piers E F Daubeney, Amrit Lota, Katie Linter, Sujeev Mathur, Tara Bharucha, Khoon Li Kok, Satish Adwani, Caroline B Jones, Zdenka Reinhardt, Rumana Z Omar, Juan Pablo Kaski

**Affiliations:** 1 Centre for Inherited Cardiovascular Diseases, Great Ormond Street Hospital, Great Ormond Street, London, UK; 2 Institute of Cardiovascular Sciences University College London, London, UK; 3 ERN GUARD-HEART (European Reference Network for Rare and Complex Diseases of the Heart); 4 Department of Statistical Science, University College London, London, UK; 5 Department of Paediatric Cardiology, Royal Hospital for Children, Glasgow, UK; 6 Department of Paediatric Cardiology, University Hospitals Bristol NHS Foundation Trust, Bristol, UK; 7 Department of Paediatric Cardiology, Birmingham Women and Children’s NHS Foundation Trust, Birmingham, UK; 8 Department of Paediatric Cardiology, University Hospital of Wales, Cardiff, UK; 9 Department of Paediatric Cardiology, Leeds Teaching Hospital NHS Trust, Leeds, UK; 10 Department of Paediatric Cardiology, Royal Brompton Hospital, National Heart and Lung Institute, Imperial College London, London, UK; 11 Department of Paediatric Cardiology, University Hospitals of Leicester, Leicester, UK; 12 Department of Paediatric Cardiology, Evelina London Children’s Hospital, Guys and St Thomas’ NHS Foundation Trust, London, UK; 13 Department of Paediatric Cardiology, University Hospital Southampton NHS Foundation Trust, Southampton, UK; 14 Department of Paediatric Cardiology, Oxford University Hospitals NHS Foundation Trust, Oxford, UK; 15 Department of Paediatric Cardiology, Alder Hey Children’s Hospital, Liverpool, UK; 16 Department of Paediatric Cardiology, The Freeman Hospital, Newcastle, UK

**Keywords:** Hypertrophic cardiomyopathy, Sudden death, Children, Risk, ESC Guidelines

## Abstract

**Aims:**

Sudden cardiac death (SCD) is the most common cause of death in children with hypertrophic cardiomyopathy (HCM). The European Society of Cardiology (ESC) recommends consideration of an implantable cardioverter-defibrillator (ICD) if two or more clinical risk factors (RFs) are present, but this approach to risk stratification has not been formally validated.

**Methods and results:**

Four hundred and eleven paediatric HCM patients were assessed for four clinical RFs in accordance with current ESC recommendations: severe left ventricular hypertrophy, unexplained syncope, non-sustained ventricular tachycardia, and family history of SCD. The primary endpoint was a composite outcome of SCD or an equivalent event (aborted cardiac arrest, appropriate ICD therapy, or sustained ventricular tachycardia), defined as a major arrhythmic cardiac event (MACE). Over a follow-up period of 2890 patient years (median 5.5 years), MACE occurred in 21 patients (7.5%) with 0 RFs, 19 (16.8%) with 1 RFs, and 3 (18.8%) with 2 or more RFs. Corresponding incidence rates were 1.13 [95% confidence interval (CI) 0.7–1.73], 2.07 (95% CI 1.25–3.23), and 2.52 (95% CI 0.53–7.35) per 100 patient years at risk. Patients with two or more RFs did not have a higher incidence of MACE (log-rank test *P* = 0.34), with a positive and negative predictive value of 19% and 90%, respectively. The C-statistic was 0.62 (95% CI 0.52–0.72) at 5 years.

**Conclusions:**

The incidence of MACE is higher for patients with increasing numbers of clinical RFs. However, the current ESC guidelines have a low ability to discriminate between high- and low-risk individuals.

**What’s new?**

- The incidence of an arrhythmic event increases with additional clinical risk factors, however, the current European Society of Cardiology (ESC) 2014 guideline for risk stratification in childhood hypertrophic cardiomyopathy (HCM) has a low ability to discriminate between high- and low-risk individuals.
- The positive predictive value of the ESC treatment threshold is low, leading to unnecessary implantable cardioverter-defibrillator implantation in many patients.
- Large-scale, multi-centre, collaborative approaches to risk stratification in childhood HCM are needed to develop robust risk stratification algorithms.

## Introduction

Hypertrophic cardiomyopathy (HCM) is the second commonest cardiomyopathy occurring during childhood, with an estimated annual incidence of 0.24–0.47 per 100 000 and prevalence of 2.7 per 100 000.^1–3^ Early reports suggested a very poor prognosis in childhood HCM,^4^ but more recent population-based and registry studies have reported annual mortality rates between 1% and 2.5%.^5–8^ The most common cause of mortality outside of infancy is sudden cardiac death (SCD),^6^ making risk stratification for arrhythmic events one of the cornerstones of the management of children with HCM. Despite this, identification of those at high risk of SCD remains a challenge, and there is currently a lack of evidence to support current risk stratification algorithms for SCD in childhood HCM. The European Society of Cardiology (ESC) guidelines^9^ recommend the use of four clinical risk factors (RFs) to stratify risk in children with HCM: unexplained syncope, family history of SCD, extreme left ventricular hypertrophy (LVH), and non-sustained ventricular tachycardia (NSVT) on ambulatory electrocardiography (ECG) recordings. An implantable cardioverter-defibrillator (ICD) is recommended if two or more clinical RFs are present. However, the efficacy of this approach to risk stratification has not previously been validated. Although ICDs have been shown to be effective at aborting malignant arrhythmias in children with HCM,^10^^,^^11^ these younger patients experience a higher rate of complications than adults,^10^ reinforcing the need to robustly identify individuals most likely to benefit from device implantation. This study sought to perform the first validation of the 2014 ESC guidelines in a cohort of childhood HCM from the UK.

## Methods

### Patients

Longitudinal clinical and outcome data were collected from a retrospective multi-centre cohort of children diagnosed with HCM in the UK between 1980 and 2017. Complete data were available from 13 out of the 14 UK specialist paediatric cardiac centres. Patients aged 16 years or younger meeting the diagnostic criteria for HCM were eligible for inclusion. This included patients with phenocopies of sarcomeric HCM (e.g. RASopathies, inborn errors of metabolism, or neuromuscular diseases). The diagnosis of HCM was made in the presence of left ventricular wall thickness >2 SD above the body surface area (BSA)-corrected population mean (*z*-score ≥+2), which could not be solely explained by abnormal loading conditions.^9^ Data on the clinical presentation and survival of the entire cohort have been recently published.^8^ In the present study, patients with a history of sustained ventricular arrhythmias or aborted SCD were excluded from analysis, as they meet criteria for secondary prevention ICD implantation. Eligible patients were identified by the principle investigator at each site using multiple sources, including medical databases and echocardiography log books. Where available, the reasons for diagnosis included family screening, symptoms, and incidental findings of abnormal ECG or heart murmur.

### Data collection

A single researcher (G.N.) visited each site to assist with data collection and confirm diagnostic classification. Anonymized, non-invasive clinical data were collected from baseline evaluation, including demographics, aetiology, symptoms, family history, resting and ambulatory ECG, two-dimensional Doppler and colour transthoracic echocardiography, and device interrogation of arrhythmic events. Aetiology was classified as non-syndromic in the absence of a diagnosis of a RASopathy syndrome (Noonan or other malformation syndrome), neuromuscular disease (including Friedreich’s ataxia), or inborn error of metabolism.

In accordance with the 2014 ESC guidelines,^9^ RFs for an arrhythmic event were assessed at baseline and comprised: NSVT—defined as three or more consecutive ventricular beats at a rate of >120 beats/min with a duration of <30 s on ambulatory ECG recordings; severe LVH—defined as a maximal left ventricular wall thickness (MLVWT) ≥30 mm or ≥6 SD above the population mean for BSA; unexplained syncope—defined as a transient loss of consciousness with no identifiable cause; and family history of SCD—defined as SCD in a first degree relative <40 years of age or SCD in a relative with confirmed HCM at any age.

### Outcomes and follow-up

All patients were routinely evaluated every 6–12 months up to 1 October 2017. The primary patient outcome, taken from last clinic appointment, was a composite outcome of SCD or an equivalent event (aborted cardiac arrest, appropriate ICD therapy, or sustained ventricular tachycardia associated with haemodynamic compromise or syncope), defined as a major arrhythmic cardiac event (MACE).

### Statistical analysis

Body surface area was calculated from height and weight.^12^ Left ventricular wall thickness measurements are expressed in millimetres and as *z*-scores relative to the distribution of measurements vs. BSA in normal children, using previously published normal values.^13^ Normally distributed continuous variables are described as mean ± standard deviation. Skewed data are described as median [interquartile range (IQR)]. Kaplan–Meier survival curves were used to obtain estimates of the incidence of a MACE and Poisson exact confidence intervals calculated. Due to small patient numbers, patients with two or more RFs were combined and group differences in survival were assessed using the log-rank test. A significance level of 0.05 was used for all comparisons.

**Figure 1:**
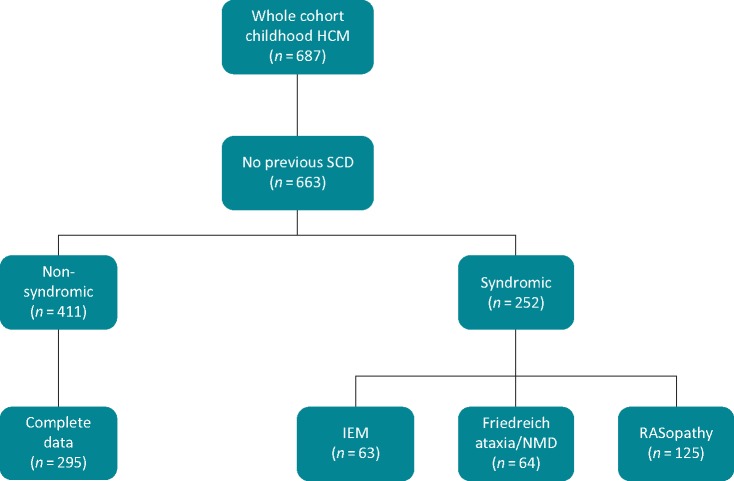
Flow chart showing aetiology of HCM in whole cohort. HCM, hypertrophic cardiomyopathy; IEM, inborn error of metabolism; NMD, neuromuscular disorder; SCD, sudden cardiac death.

For the purpose of analysis, patients with syndromic HCM were excluded as they are a heterogeneous population with variable long-term outcomes, which are partly determined by the underlying aetiology. However, as the ESC guidelines do not specifically exclude patients with non-sarcomeric disease, separate analyses were also performed including these patients (Supplementary material online) (*Figure 1*). The ESC guidelines assign each clinical RF equal weight and the overall clinical risk profile is calculated as the sum of RFs present. For the purpose of assessing the discriminatory ability of the ESC algorithm, missing data for RFs were coded as absent to reflect the use of the guideline in clinical practice.^14^ As this may be a source of bias, a complete case analysis was also performed (Supplementary material online). The RF profile was considered as a continuous score ranging from 0 (when all RFs were absent) to 4 (when all RFs were present) and the C-statistic at 1 and 5 years was estimated. The receiver operating characteristic curve was constructed by plotting the sensitivity against (1 − specificity) for each possible prognostic value (e.g. ≥0, ≥1, ≥2, ≥3, or ≥4 RFs) and then calculating the area under the curve. The positive predictive value (PPV) of a particular RF profile was calculated by dividing (sensitivity × prevalence) by ((sensitivity × prevalence) + (1 − specificity) × (1 − prevalence)) and expressed as a percentage. The negative predictive value (NPV) was calculated by dividing (specificity × (1 − prevalence)) by ((1 − sensitivity × prevalence) + (specificity × (1 − prevalence)) and expressed as a percentage. Statistical analysis was performed using StataCorp. 2015. Stata Statistical Software: Release 14. College Station, TX, USA: StataCorp LP.

### Ethics

Ethical committee approval was obtained at each participating site. The study complies with the principles of Good Clinical Practice and the Declaration of Helsinki.

## Results

From the total cohort of 687 patients,^8^ 411 patients had non-syndromic HCM, diagnosed aged 16 years or younger, and without a previous history of sustained ventricular tachycardia or aborted cardiac arrest (*Figure 1*). Baseline clinical characteristics are shown in *Table 1*. Median age at diagnosis was 10 years (IQR 3–13); 60 patients (14.6%) presented under the age of 1 year. Pathogenic mutations in sarcomeric protein genes were reported in 94 patients (23%). One hundred and ten patients (26.8%) had an ICD implanted during follow-up for primary prevention [*n* = 99 (90%)] and secondary prevention [11 (10%)]. Of those with a secondary prevention device, the indication for ICD implantation was an aborted SCD in nine patients and sustained VT on ambulatory ECG monitoring in two.

**Table 1:** Clinical characteristics of non-syndromic study population at baseline evaluation

Age (years), median (IQR)	10 (3–13)
Infant	60 (14.6%)
1–<6 years	73 (17.8%)
6–<12 years	113 (27.5%)
12–<16 years	165 (40.1%)
Male gender	271 (66%)
NYHA III/IV (*n* = 410)	15 (3.7%)
Maximal wall thickness at baseline (mm), mean (range) (*n* = 398)	17.0 (6–48)
Left ventricular outflow tract obstruction (>30 mmHg) (*n* = 374)	104 (27.8%)
Procedures during study period	
Myectomy	37 (8.9%)
ICD	110 (26.8%)
Pacemaker	23 (5.6%)

*n* = 411 unless otherwise indicated.

ICD, implantable cardioverter-defibrillator; IQR, interquartile range; NYHA, New York Heart Association.

### Prevalence of European Society of Cardiology clinical risk factors

The prevalence at baseline of each of the four major clinical RFs described in the 2014 ESC guidelines is shown in *Table 2*. Two hundred and eighty-two patients (68.7%) had no traditional clinical RFs, 113 (27.5%) had a single clinical RF, 14 (3.4%) had two clinical RFs, and two patients (0.5%) had three clinical RFs. No patients had all four clinical RFs. Complete data for all clinical RFs were available for 295 patients (71.8%), data were missing for one or two RFs in 111 patients (27%) and 5 patients (1.2%), respectively.

**Table 2:** Prevalence of ESC clinical risk factors at baseline

	Non-syndromic only, *n* (%) (*n* = 411)
MWT ≥30 mm or *z*-score ≥6	63/398 (15.8)
NSVT	5/307 (1.6)
Unexplained syncope	30/409 (7.3)
Family history of SCD	49/409 (12)

ESC, European Society of Cardiology; MWT, maximal wall thickness; NSVT, non-sustained ventricular tachycardia; SCD, sudden cardiac death.

### Arrhythmic events and clinical risk factor profile

Median length of follow-up was 5.5 years (IQR 2.4–10.6). Over a total follow-up period of 2890 patient years, 43 patients had a MACE with an event rate of 1.5 per 100 patient years at risk. The MACE was SCD in 17 (4.1%); aborted cardiac arrest in 9 (2.2%); appropriate ICD discharge in 12 (2.9); and sustained VT associated with haemodynamic compromise in 5 (1.2%). Sustained VT was documented on ICD download (*n* = 3) or an implantable ambulatory ECG device (*n* = 2).

**Figure 2:**
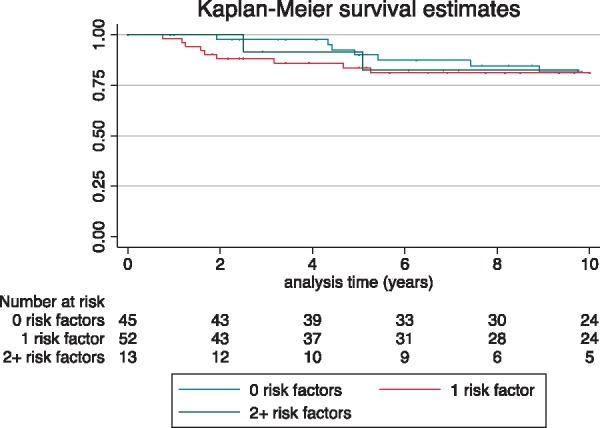
Kaplan–Meier survival curve showing event-free survival from MACE over follow-up for different clinical risk factor profiles. Log-rank test 0.8993. MACE, major arrhythmic cardiac event.

A MACE occurred in 21 patients (7.5%) with no clinical RFs; 19 patients (16.8%) with one clinical RF; and 3 patients (18.8%) with two or more clinical RFs (*Table 3*). Event-free survival at 5 years for patients with 0, 1, or ≥2 clinical RFs was 93.8% [95% confidence interval (CI) 89.3–96.5%], 87.6% (95% CI 79.1–92.8%), and 86.7% (56.4–96.5%), respectively (*Figure 2*)*.* The corresponding incidence rates are shown in *Table 3*.

**Table 3:** Incidence of a MACE by clinical risk profile

	Non-syndromic only (*n* = 411)	
Number of clinical risk factors	MACE, *n* (%)	Incidence rate/100 patient years (95% CI)
0	21 (7.5)	1.13 (0.70–1.73)
1	19 (16.8)	2.07 (1.25–3.23)
≥2	3 (18.8)	2.52 (0.53–7.35)

CI, Poisson exact 95% confidence interval; MACE, major arrhythmic cardiac event.

Of the 113 patients with a single ESC RF, 49 (44%) had severe LVH; 22 (20%) unexplained syncope; 40 (35%) a family history of SCD; and 2 (2%) NSVT. The incidence of MACE in patients with a single clinical RF is shown in *Table 4.* The PPV and NPV of a single clinical RF for a MACE was 17.1% and 92.6%, respectively.

**Table 4:** Incidence of a MACE in patients with non-syndromic HCM and a single risk factor

	*N* (%)	Length of FU (years), median (IQR)	MACE, *n* (%)	Incidence rate/100 patient years (95% CI)	5-Year cumulative incidence of MACE
MWT ≥30mm or *z*-score ≥6	49 (44%)	6.3 (3.08–12)	9 (16%)	1.9 (0.83–3.80)	9.3 (3.57–22.9)
Unexplained syncope	22 (20%)	4.1 (1.83–9.42	6 (27%)	4.2 (1.53–9.1)	27.6 (12.2–54.9)
Family history of SCD	40 (34%)	7.1 (2.5–12.8)	5 (12.5%)	1.5 (0.5–3.59)	8.8 (2.9–25.1)
NSVT	2 (2%)	17.9 (15.33–20.58)	0		

*n* = 113.

CI, Poisson exact 95% confidence interval; FU: follow-up; HCM, hypertrophic cardiomyopathy; IQR, interquartile range; MACE, major arrhythmic cardiac event; MWT, maximal wall thickness; NSVT, non-sustained ventricular tachycardia; SCD, sudden cardiac death.

**Figure 3:**
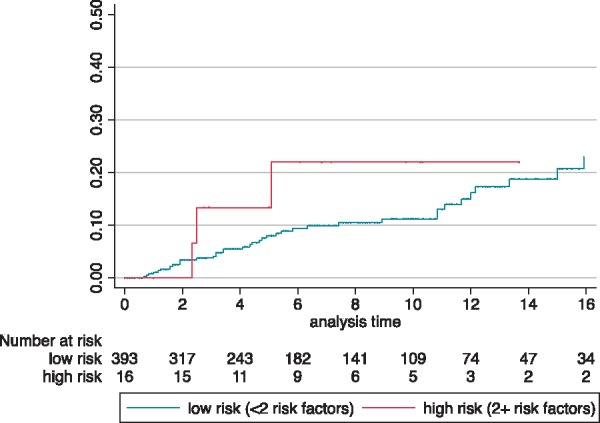
Cumulative incidence of MACE for patients at low (<2 clinical risk factors) or high (≥2 clinical risk factors) risk for SCD according to ESC treatment threshold. Log-rank test *P *=* *0.34. ESC, European Society of Cardiology; MACE, major arrhythmic cardiac event; SCD, sudden cardiac death.

Patients classified as ‘high risk’ according to the ESC guidelines (≥2 clinical RFs) did not have a higher incidence of MACE compared to ‘low risk’ patients (log-rank test *P* = 0.34) (*Figure 3*).

### Discrimination performance of the 2014 European Society of Cardiology risk stratification guidelines

**Figure 4:**
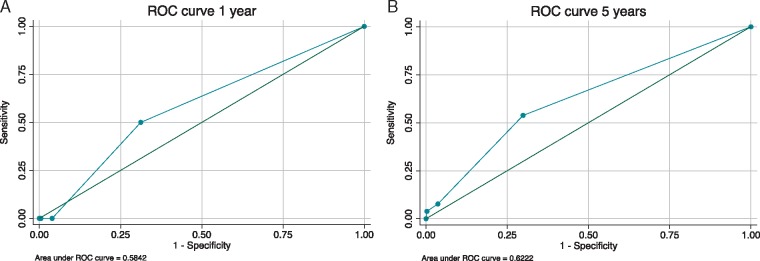
Receiver operating characteristic curves for the ESC guidelines at (*A*) 1 year and (*B*) 5 years, respectively. ESC, European Society of Cardiology; ROC: Receiver operating characteristic.

The C-statistic, which represents the probability of correctly distinguishing between high and low risk patients using the 2014 ESC clinical risk profile, was 0.58 (95% CI 0.31–0.86) at 1 year and 0.62 (95% CI 0.52–0.72) at 5 years (*Figure 4*)*.* The PPV and NPV of the 2014 ESC treatment thresholds for primary prevention ICD implantation (≥2 clinical RFs) at 5-year follow-up and end of follow-up were 12.5%/93.9% and 18.8/89.9%, respectively. A complete case analysis (*n* = 295) did not improve the discrimination performance of the guideline with a PPV and NPV of 23.1% and 89%, respectively, and C-statistic of 0.63 (95% CI 0.54–0.72) (Supplementary material online). A sensitivity analysis including syndromic patients (*n* = 663) did not improve the discrimination performance of the guideline with a PPV and NPV of 23.5% and 92.9%, respectively, with a c-statistic of 0.63 (95% CI 0.557–0.703).

## Discussion

This study is the first external validation of the current ESC guidelines for risk stratification in childhood HCM. We show that the current guidelines have only a modest ability to discriminate between patients with non-syndromic HCM at a low or high risk of an arrhythmic event, with a C-statistic of 0.62 at 5 years.

### Risk factors for sudden cardiac death in childhood hypertrophic cardiomyopathy

The majority of patients (69%) in this cohort had none of the traditional clinical RFs for SCD historically used for risk stratification in adults, and only a small number of patients met the ESC threshold for ICD implantation (16 patients, 3.9%). However, the overall rate of arrhythmic events was higher in childhood disease compared to adulthood (1.1 vs. 0.8%), particularly for those with non-syndromic disease (1.5 vs. 0.8%). This suggests that additional, or alternative, clinical RFs for SCD are important in childhood onset disease. Indeed, although a large number of potential RFs for SCD in childhood HCM have been reported in the literature, the lack of consistent definitions and sufficiently large, suitably designed population studies means that the evidence for individual RFs, including those endorsed by the ESC guidelines, is not robust. A meta-analysis of published clinical RFs for SCD in childhood HCM^15^ identified four clinical RFs that are likely to be important for risk stratification in children: previous aborted cardiac arrest, NSVT, extreme LVH, and unexplained syncope. Importantly, a family history of SCD, which is included in the ESC guidelines, was not identified as a major RF as it was only associated with SCD in one^11^ out of seven included studies. Possible explanations for this include: a higher prevalence of de novo mutations in childhood HCM; insufficient reporting of family history in included studies; or lack of adjustment for family linkage. In addition, although LVH has been shown to be associated with SCD in several studies, only one reported a significantly increased risk associated with an MLVWT ≥30 mm or ≥6 SD above population mean for BSA,^16^ which is the definition endorsed by the guidelines. A systematic assessment of clinical RFs in childhood HCM is therefore required to identify the most useful clinical features to predict risk.

### Discriminatory power of current guidelines for risk stratification in childhood

As each traditional clinical RF included in the ESC guidelines is a marker of more severe disease, it is perhaps not surprising that the risk of an event increased incrementally with summative RFs. However, the incidence rate did not significantly differ between those classified by the guideline as high risk or low risk, meaning that the ability of the guideline threshold to distinguish between those patients at high and low risk was limited. Although the arrhythmic incidence rate was lowest for patients with no clinical RFs, the majority of events (63%) occurred in this low-risk group. Furthermore, the low PPV (18.8%) means that the majority of patients in whom an ICD is recommended will not experience an event but will be exposed to the potential complications associated with ICD implantation. In this cohort of 110 patients with an ICD, only 11% had an appropriate discharge to treat a malignant arrhythmia. The low number of patients receiving appropriate therapy likely highlights the difficulties in identifying patients at increased risk. Indeed, over half (56%) of MACE occurred in patients not judged to be at increased risk by clinicians who therefore did not undergo ICD implantation. Application of the ESC clinical guideline did not improve patient identification with 93% of arrhythmic events occurring in patients with 0 or 1 clinical RFs. This is a particular problem for childhood HCM, as it has been shown that younger patients are at higher risk of ICD-related complications^10^ and will also be exposed to these risks for a longer period of time. The availability of subcutaneous ICDs, which avoid the need for intravascular or intracardiac leads, may reduce the complication burden in this group of patients.

The current guidelines do not specifically exclude patients with syndromic HCM (RASopathy syndrome, neuromuscular disease, or inborn error of metabolism) and excluding these patients from the analysis did not improve the discriminatory power of the guidelines. Of note, current North American guidelines^17^ make use of the same traditional RFs (unexplained syncope, family history of SCD, and extreme LVH), although recommend that an ICD is considered reasonable in the presence of a single RF only. The presence of a single RF was associated with an increased incidence of an arrhythmic event (17% within 5 years), but the discriminatory power of this threshold for ICD was similarly poor (PPV 17.1%; NPV 92.6%). The small number of patients with a single RF prevents a meaningful comparison between the risk of MACE for each individual RF as the calculated confidence intervals are wide (*Table 4*). However, the high NPV of the ESC treatment threshold (89.9%) supports the notion that patients with 0 or 1 clinical RF are at a low risk for ventricular arrhythmias and can be reassured. Large multi-centre collaborative studies are required to investigate the role of individual RFs in childhood HCM.

### Future directions

Childhood HCM is recognized to be a heterogeneous disease.^5^^,^^6^ In our UK cohort, 37% of patients had either a RASopathy syndrome, neuromuscular disease, or inborn error of metabolism, and age of diagnosis ranged from 1 to 16 years of age.^8^ Current guidelines do not account for this heterogeneity, providing relative risks for an arrhythmic event for non-homogenous groups rather than individualized estimates. This approach to risk stratification necessarily converts continuous variables [e.g. maximal wall thickness (MWT)] into a binary variable (e.g. MWT < 30 mm or MWT ≥ 30 mm), the clinical validity of which, particularly during childhood which is a period of significant somatic growth, may be questioned. In agreement with our findings, this approach to risk stratification has been shown to have a low predictive power in adult HCM patients, with unnecessary ICD implantation in a large number of patients.^14^ As a result, more recently, the ESC endorsed the use of an SCD risk prediction model (HCM Risk-SCD) which provides an individualized estimate of 5-year SCD risk.^18^ External validation of this approach to risk stratification has shown that this model has an improved discriminatory power (c-index 0.7),^19^ although other studies have reported that it underestimates risk.^20^ Nonetheless, it is not currently validated for use in patients under the age of 16 years of age. The ability to provide an individualized estimate for the risk for SCD in childhood HCM would be of significant benefit in clinical practice.

### Limitations

This study is limited by inherent problems of retrospective studies, in particular missing or incomplete data. Although the classification of missing RF data as absent is a source of potential bias, this reflects real world practice where absence of a RF is often taken to mean absence of risk. This approach to analysis replicates that used by other retrospective validation studies in adult HCM (Ref.^14^) in which only 78% of patients had a complete RF dataset. The higher percentage of missing data in our cohort likely reflects difficulties in obtaining certain investigations (e.g. ambulatory ECGs) in very young patients but may have resulted in an under-estimation of the prevalence of clinical RFs (particularly arrhythmic events). Limiting data analysis to those patients with complete data did not alter the overall results of the study and did not improve the discriminatory performance of the guideline (Supplementary material online).

As childhood HCM is a rare disease and SCD a relatively rare outcome, despite this study containing one of the largest populations of childhood HCM to date, there were a small number of events and small number of patients classified as ‘high risk’ according to the ESC guidelines. This reduced our power to detect statistically significant differences and, as a result, the reported confidence intervals are wide. The relatively short mean follow-up time of 5.5 years (range 6 months–25 years) could partly explain the low number of events. However, it is important to note that the current guidelines do not provide an estimate of risk at a particular time point. Relatively little is known about the sample size requirements for external validation of prediction models, although a sample containing at least 100 events and 100 non-events has been suggested.^21^ Using published rates of arrhythmic events in childhood HCM of 1.5/100 patient years^8^ with a median follow-up of 5 years, this would require over 1300 patients assuming no dropout during the 5-year follow-up period. Such a sample size would be challenging to achieve for a rare condition with a reported prevalence of childhood HCM below 3/100 000. Furthermore, this study only includes RF assessment findings from the initial clinical evaluation, in keeping with current ESC recommendations^9^ and previous adult studies,^14^ and does not assess disease progression and potential changing risk profiles with time. Large-scale, prospective, multi-centre studies, or registries with standardized investigation protocols are needed to overcome and address these challenges in risk stratification for childhood HCM.

In this cohort, only 23% of non-syndromic patients had a confirmed pathogenic mutation in a cardiac sarcomere protein gene. This is almost certainly an underestimate of the true prevalence as previous studies have shown that most cases of HCM are caused by sarcomeric mutations, even in young children.^22^ This finding likely reflects a lack of consistent and systematic genetic testing in this cohort. Although the long period of recruitment may result in changes in assessment and therapies influencing the findings in this study, we have previously shown no era effect in survival in this cohort.^8^

## Conclusions

This study is the first validation of the 2014 ESC risk stratification guidelines for childhood HCM. The results suggest that the current guidelines have a limited ability to distinguish between high- and low-risk patients. Although the incidence of an arrhythmic event increases with additional RFs, the PPV of the treatment threshold is low with unnecessary ICD implantation in many patients. Large-scale international collaborative studies are required to overcome the challenges of small patient numbers inherent to rare diseases.

## Funding

This work was supported by the British Heart Foundation (grant number FS/16/72/32270 to G.N. and J.P.K.). E.F. and J.P.K. are supported by Max’s Foundation and Great Ormond Street Hospital Children’s Charity. This work is (partly) funded by the NIHR GOSH BRC. The views expressed are those of the author(s) and not necessarily those of the NHS, the NIHR, or the Department of Health. R.Z.O. and T.D. work at University College London Hospitals/University College London who received a proportion of funding from the United Kingdom Department of Health’s National Institute for Health Research Biomedical Research Centres funding scheme. The views expressed are those of the authors and not necessarily those of the NHS, the NIHR, or the Department of Health.

**Conflict of interest:** none declared.

## Supplementary Material

euz118_Supplementary_Data
