# Supplementary material for: A validation study of the European Society of Cardiology guidelines for risk stratification of sudden cardiac death in childhood hypertrophic cardiomyopathy
Source: Europace. 2019 Jun 1;21(10):1559–65. doi: 10.1093/europace/euz118 (PMC6788212; doi:10.1093/europace/euz118)
Supplement: euz118_Supplementary_Data [file euz118_supplementary_data.docx]

**Supplementary data: Complete case analysis**

*Discrimination performance of the 2014 ESC risk stratification guidelines in patients with non-syndromic HCM and complete data:*

In 295 patients (n=72%) with non-syndromic HCM the prevalence of ESC clinical risk factors is shown in *supplementary table 1*. Over a total follow up period of 2012 patient years, 34 patients had a MACE [SCD in 13 (38%); aborted cardiac arrest in 5 (14.7%); appropriate ICD discharge in 11 (32.4%); and sustained VT in 5 (14.7%)], with an event rate of 1.69 per 100 years at risk. Freedom from MACE at 5 years for patients with 0, 1, or ≥2 clinical risk factors was 94% (95% CI 87.5-96.8%), 88% (95% CI 78.5-93.3%), and 85% (95% CI 51.2-95.9%), respectively. Corresponding incidence rates are show in *supplementary table 2*. The PPV and NPV of the 2014 treatment threshold for primary prevention ICD implantation (≥2 clinical risk factors) were 23.1% and 89% respectively, with a C-statistic of 0.63 (95% CI 0.54-0.72)

*Discrimination performance of the 2014 ESC risk stratification guidelines in patients with syndromic and non-syndromic HCM:*

In a cohort of 663 patients with childhood HCM*:* 411 patients (62%) had non-syndromic HCM; 125 patients (19%) had a RASopathy syndrome; 64 patients (10%) had Friedreich’s ataxia or another neuromuscular disorder; and 63 (10%) had an inborn error of metabolism (*Figure 1)*. The prevalence of clinical risk factors is shown in *Table 2.*

Over a median length of follow-up of 5.3 years (IQR 2.33-10), 50 patients had a MACE [SCD in 19 (2.9%); aborted cardiac arrest in 10 (1.5%); appropriate ICD discharge in 13 (2%); sustained VT in 8 (1.2%)], with an event rate of 1.1 per 100 patient years at risk. The incidence of a MACE by clinical risk factor profile is shown in *table 3.*  The PPV and NPV of the 2014 ESC treatment thresholds for primary prevention ICD implantation (≥2 clinical risk factors) were 23.5% and 92.9%, respectively, with a c-statistic of 0.63 (95% CI 0.557 – 0.703).

**Supplementary table 1: Prevalence of ESC clinical risk factors at baseline in patients with complete case analysis**

|  | Complete case analysis on-syndromic, n (%) [n=295] | Whole cohort, n (%)  [n= 663] |
| --- | --- | --- |
| MWT ≥ 30mm or Z score ≥ 6 | 51 (17%) | 73/623 (11%) |
| NSVT | 5 (1.7%) | 6/461 (0.9%) |
| Unexplained syncope | 24 (8.4%) | 36/661 (5.4%) |
| Family history of SCD | 42 (14.2%) | 54/659 (8.1%) |

MWT= maximal wall thickness, NSVT = Non-sustained Ventricular Tachycardia, SCD = sudden cardiac death

**Supplementary table 2: Incidence of a MACE by clinical risk profile**

|  | Complete case analysis non-syndromic only n=295 | | Whole cohort n=663 | |
| --- | --- | --- | --- | --- |
| Number of clinical risk factors | MACE (n, %) | Incidence rate/100 patient years (95% CI) | MACE (n,%) | Incidence rate/100 patient years (95% CI) |
| **0** | 14 (7.4%) | 1.23 (0.68-2.07) | 27 (5.3%) | 0.83  (0.54-1.20) |
| **1** | 17 (18%) | 2.20 (1.28 – 3.52) | 19 (14.3%) | 1.75  (1.05-2.73) |
| **≥2** | 3 (23%) | 2.84 (0.58 – 8.30) | 4 (24%) | 3.27  (0.89-8.37) |

CI = Poisson exact 95% confidence interval

In 295 patients (n=72%) with non-syndromic HCM and complete data over a total follow up period of 2012 patient years, 34 patients had a MACE [SCD in 13 (38%); aborted cardiac arrest in 5 (14.7%); appropriate ICD discharge in 11 (32.4%); and sustained VT in 5 (14.7%)], with an event rate of 1.69 per 100 years at risk. Freedom from MACE at 5 years for patients with 0, 1, or ≥2 clinical risk factors was 94% (95% CI 87.5-96.8%), 88% (95% CI 78.5-93.3%), and 85% (95% CI 51.2-95.9%), respectively. The PPV and NPV of the 2014 treatment threshold for primary prevention ICD implantation (≥2 clinical risk factors) were 23.1% and 89% respectively, with a C-statistic of 0.63 (95% CI 0.54-0.72).
